# The Role of Phase Angle in Non-Invasive Fluid Assessment in Dogs with Patent Ductus Arteriosus: A Novel Method in Veterinary Cardiology

**DOI:** 10.3390/vetsci12101007

**Published:** 2025-10-17

**Authors:** Zongru Li, Ahmed Farag, Ahmed S. Mandour, Tingfeng Xu, Kazuyuki Terai, Kazumi Shimada, Lina Hamabe, Aimi Yokoi, Shujun Yan, Ryou Tanaka

**Affiliations:** 1 Department of Veterinary Surgery, Faculty of Veterinary Medicine, Tokyo University of Agriculture and Technology, Fuchu 183-8509, Japanterai.kazuyuki.dvm@gmail.com (K.T.);; 2 Department of Animal Medicine (Internal Medicine), Faculty of Veterinary Medicine, Suez Canal University, Ismailia 41522, Egypt

**Keywords:** bioelectrical impedance analysis, patent ductus arteriosus, phase angle, pulmonary fluid balance, veterinary cardiology

## Abstract

**Simple Summary:**

Patent ductus arteriosus (PDA) is a congenital heart defect in dogs that causes left-to-right shunting, increasing pulmonary blood flow and placing excess load on the left heart. This can lead to pulmonary edema and ventricular remodeling. Monitoring fluid imbalance is essential for staging the disease and managing recovery, but conventional tools such as echocardiography or blood markers do not provide real-time, region-specific information. This study evaluated phase angle (PhA), derived from bioelectrical impedance analysis (BIA), as a non-invasive marker of thoracic fluid status in dogs with PDA. Thoracic PhA at 5 kHz was significantly reduced in PDA dogs, strongly correlated with pulmonary velocity, and moderately correlated with end-diastolic volume, reflecting extracellular fluid accumulation. After surgical correction, PhA values progressively increased, indicating recovery of thoracic fluid balance. Regional comparisons showed thoracic PhA was more sensitive than trunk or abdominal values, while associations with structural echocardiographic indices were limited. Overall, low-frequency thoracic PhA appears to be a practical tool for assessing fluid dynamics, monitoring treatment response, and supporting clinical decision-making in veterinary cardiology.

**Abstract:**

Background: Patent ductus arteriosus (PDA) in dogs causes persistent left-to-right shunting, leading to pulmonary overcirculation, left heart volume overload, and potential congestive heart failure. Accurate assessment of fluid imbalance is essential but challenging with conventional echocardiography or biomarkers. Phase angle (PhA), derived from bioelectrical impedance analysis (BIA), may serve as a non-invasive marker of extracellular fluid distribution and cellular integrity. Objectives: This study aimed to evaluate PhA as an indicator of thoracic fluid imbalance in dogs with PDAby analyzing its correlation with pulmonary velocity (PV) and end-diastolic volume (eV), as well as its responsiveness to surgical correction. In addition, we assessed the relationships between PhA and echocardiographic structural indices (LA/Ao, TDI Sep E/Em, TDI Lat E/Em) and examined the influence of the measurement region. Methods: PhA was measured at 5, 50, and 250 kHz in 30 PDA-affected and 15 healthy dogs, with electrode placement across thorax, trunk, and abdomen. Echocardiography evaluated PV, eV, and PDA-specific structural parameters. Results: Thoracic PhA at 5 kHz was significantly reduced in PDAdogs, strongly correlated with PV and moderately with eV. Postoperative measurements showed progressive PhA recovery. Only TDI Lat E/Em correlated with mid-frequency PhA, while other structural indices showed minimal association. Thoracic PhA was lower than trunk or abdominal values, indicating that thoracic measurements may better capture localized extracellular fluid changes in PDAcompared with other regions. Conclusion: Thoracic PhA at 5 kHz effectively reflects extracellular fluid changes in PDA, complements structural echocardiography, and tracks postoperative fluid normalization. Its non-invasive nature supports clinical utility for monitoring hemodynamic burden and therapeutic response.

## 1. Introduction

Patent ductus arteriosus (PDA) is one of the most common congenital cardiac anomalies in dogs, caused by postnatal persistence of the ductus arteriosus and abnormal communication between the aorta and pulmonary artery [1,2,3]. This left-to-right shunting leads to pulmonary overcirculation, volume overload of the left heart, and, if untreated, progressive left-sided heart failure (LHF) [4,5]. PDA accounts for a considerable proportion of congenital heart defects in dogs, with a recognized breed predisposition in poodles, Shetland sheepdogs, and Chihuahuas [6]. Because of its progressive and potentially fatal nature, early diagnosis and intervention are essential to prevent adverse remodeling and pulmonary vascular complications.

Accurate assessment of PDA severity and treatment response is central to clinical management. Conventional methods such as radiography, echocardiography, and circulating biomarkers (BNP, NT-pro BNP) provide important information but have clear limitations [7]. Radiography detects cardiomegaly and congestion but cannot quantify real-time fluid distribution. Echocardiography is indispensable for assessing shunt flow and cardiac loading but does not directly evaluate systemic fluid balance. Biomarkers reflect myocardial stress but cannot distinguish intra- from extracellular fluid [2,3,4,8,9]. These constraints highlight the need for new, quantitative approaches to evaluate fluid status in veterinary patients.

In human medicine, phase angle (PhA) derived from bioelectrical impedance analysis (BIA) is recognized as a non-invasive biomarker of cellular health and fluid distribution [10,11]. It has been validated as a prognostic tool in heart failure, sepsis, cancer, and chronic kidney disease. In acute decompensated heart failure, PhA correlates with natriuretic peptides, hydration, and outcomes, reflecting extracellular water shifts with higher temporal and spatial sensitivity than conventional markers [8,12].

Our previous unpublished study using trunk-based BIA in dogs showed that PhA was associated with right heart failure and correlated with osmolality but did not distinguish LHF from healthy controls. This suggests that trunk measurements may have limited value in LHF. To address this, the present study investigates PDA, a representative LHF-associated disease characterized by volume overload and pulmonary overcirculation [2].

PhA is calculated from resistance (R) and reactance (Xc) using the formula PhA = arctangent (Xc/R). It reflects both cell membrane properties and the balance of intra- and extracellular fluids. Clinically, reduced PhA is linked to impaired cell integrity, loss of cell mass, and extracellular fluid expansion, making it a useful marker in conditions with inflammation, malnutrition, or fluid overload [11,13].

Pathophysiologically, we propose the sequence: LHF → elevated pulmonary capillary pressure → interstitial pulmonary edema → localized extracellular fluid accumulation in the thorax → decline in thoracic PhA. Low-frequency PhA (5 kHz), which primarily reflects extracellular water, may therefore serve as a surrogate marker of pulmonary congestion in dogs with PDA.

Although PhA is well established in human medicine, its veterinary use remains limited. Most studies have used trunk-based electrode placement, which may not capture region-specific fluid shifts. In PDA, where fluid accumulation is mainly thoracic, thoracic-focused measurements may improve sensitivity [14].

To address this gap, we evaluated thoracic PhA as a biomarker of PDA -associated fluid imbalance. We compared PDA dogs with healthy controls, assessed pre- and postoperative changes, and analyzed correlations with echocardiographic indices (eV, PV, LA/Ao, TDI Lat E/Em, TDI Sep E/Em). PhA was also measured at thoracic, trunk, and abdominal sites to assess regional variability and guide electrode placement [15]. By integrating PhA into the diagnostic framework of PDA, this study aims to establish a practical, cost-effective, and real-time approach for monitoring fluid distribution and disease progression in veterinary cardiology.

We hypothesized that thoracic PhA, especially at low frequency, would be reduced in PDA due to extracellular fluid accumulation, increase after surgical correction, and show stronger correlations with echocardiographic indices than trunk or abdominal measurements.

## 2. Materials and Methods

### 2.1. Study Design and Population

This prospective study was conducted at the Veterinary Medical Teaching Hospital, Fuchu Campus, Tokyo University of Agriculture and Technology, between March 2024 and March 2025. The study population comprised canine patients diagnosed with Patent Ductus Arteriosus (PDA) and a control group of clinically healthy dogs. Inclusion criteria for the PDA group were echocardiographic confirmation of a left-to-right shunt and clinical signs consistent with PDA (Table 1). Control dogs had no clinical or echocardiographic evidence of cardiovascular disease.

To explore the relationship between bioimpedance-derived indices and structural or functional cardiac alterations, the following echocardiographic parameters were selected: early diastolic mitral inflow velocity (eV) and peak Pulmonary Velocity (PV) [16,17,18]. eV reflects left ventricular diastolic filling and volume overload secondary to chronic shunting, whereas PV provides a direct index of pulmonary overcirculation severity.

### 2.2. Inclusion and Exclusion Criteria

Dogs were included if they met echocardiographic criteria for PDA, including continuous left-to-right shunting across the ductus arteriosus, evidence of volume overload (LA/LV enlargement), and elevated pulmonary artery flow velocity. Only cases with complete echocardiographic and clinical data were eligible. Healthy control dogs were selected from first-visit cases diagnosed with non-systemic, non-cardiac conditions such as orthopedic or ophthalmologic issues. All control animals underwent complete physical examination, echocardiography, and routine blood bio-chemistry to rule out underlying cardiac or systemic diseases, including anemia, renal dysfunction, or endocrine disorders.

Dogs were excluded if they had concurrent congenital or acquired cardiac disease other than PDA, systemic disorders known to influence hydration or fluid balance (e.g., renal, hepatic, endocrine disease), or incomplete medical or imaging records.

### 2.3. Sample Selection and Diagnostic Criteria

Dogs with PDA were enrolled following confirmation by comprehensive echocardiographic examination using two-dimensional (2D), M-mode, and color Doppler imaging. The diagnostic criteria included:

- Continuous, high-velocity turbulent flow within the main pulmonary artery on color Doppler imaging.
- Demonstration of left-to-right ductal shunting via pulsed-wave and continuous-wave Doppler.
- Left atrial and ventricular enlargement consistent with volume overload, as assessed by M-mode and 2D echocardiography.
- Elevated pulmonary artery flow velocity, reflecting excess pulmonary circulation [19,20].

Additional assessments included thoracic radiography to detect cardiomegaly and pulmonary congestion, and electrocardiography to screen for rhythm disturbances [2,6].

Healthy control dogs were included based on normal echocardiography, absence of cardiovascular clinical signs, and no prior history of systemic disease. Because fully healthy geriatric animals were rarely available, controls primarily consisted of younger dogs presented for routine wellness examinations. Consistent with previous veterinary studies [21,22], only dogs with localized, non-metabolic, and non-inflammatory conditions (e.g., fractures, inguinal hernia) were considered eligible, as these conditions were unlikely to influence hydration, fluid balance, or cellular integrity [21].

### 2.4. Anesthesia Protocol

All dogs undergoing PDA ligation were anesthetized using a standardized protocol designed for patients with congenital cardiac disease (Table 2). Premedication consisted of an opioid in combination with a benzodiazepine. Anesthesia was induced with propofol to effect, followed by endotracheal intubation, and maintained with isoflurane in oxygen using a rebreathing circuit. Cardiovascular and respiratory variables—including heart rate, arterial blood pressure, oxygen saturation, and end-tidal CO_2_—were continuously monitored throughout the procedure. This protocol was selected for its cardiovascular stability and is consistent with current recommendations for anesthetizing dogs with congenital heart disease, including PDA [6].

### 2.5. Bioelectrical Impedance Analysis

**Figure 1:**
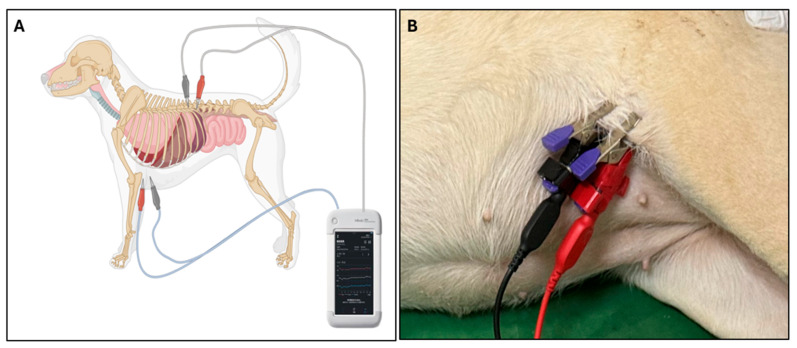
Electrode placement for thoracic bioelectrical impedance measurement. (**A**) Electrodes positioned from the left axilla to the 13th rib–spine junction to assess fluid distribution in the thoracic region. (**B**) Proper electrode–skin contact is essential for accurate measurement; in dogs with dense hair, localized hair removal is recommended to ensure optimal signal acquisition.

Phase angle (PhA) was measured using a multi-frequency bioelectrical impedance analyzer (InBody M20, InBody Co., Ltd., Seoul, Republic of Korea), which records PhA at 5, 50, and 250 kHz. Because PDA primarily causes fluid accumulation within the thoracic cavity, thoracic measurements were emphasized to better capture localized extracellular fluid changes. For thoracic measurements, electrodes were positioned from the left axilla to the spinal junction at the 13th rib, targeting regional fluid distribution within the thoracic cavity (Figure 1). PhA was assessed in both PDA and healthy control groups, and in PDA dogs both pre- and post-surgical correction, to evaluate disease-related changes and treatment effects.

**Figure 2:**
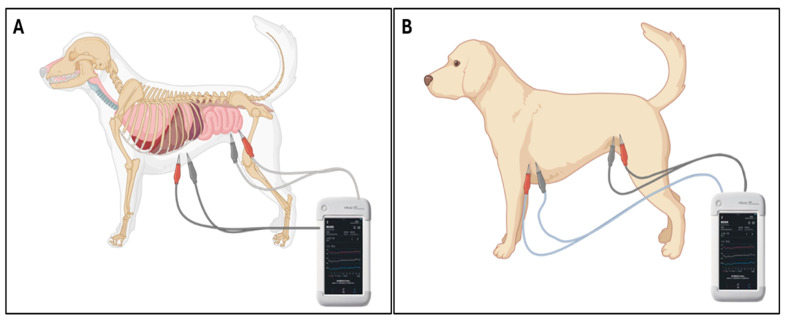
Electrode configurations for bioelectrical impedance PhA measurement. (**A**) Trunk configuration: Electrodes were positioned from the left forelimb axilla to the left hindlimb inguinal region, capturing signals across the central trunk. (**B**) Abdominal configuration: Electrodes were repositioned from the xiphoid process to the left hindlimb inguinal region, primarily targeting the abdominal cavity while minimizing thoracic signal contribution.

To investigate the regional specificity of PDA-induced fluid shifts, additional PhA measurements were obtained in the trunk and abdominal regions of PDA dogs. For trunk measurements, electrodes extended from the left axilla (cranial forelimb) to the perineal region (between hindlimbs), encompassing the entire torso. For abdominal measurements, electrodes were positioned from the caudal xiphoid process to the perineum, isolating the abdominal compartment (Figure 2). PhA values at 5, 50, and 250 kHz were recorded for each region.

Measurements were performed at 50 ms intervals for 50 repetitions (2.5 s total) and repeated at least three times. The mean of these trials was used as the representative value. All measurements were conducted without sedation or anesthesia. Veterinary ECG clip electrodes were employed to ensure consistent skin contact and stable electrode positioning.

### 2.6. Echocardiography

Standard transthoracic echocardiography was performed by an experienced cardiologist using a high-resolution ultrasound system. Examinations were conducted without sedation, and compliance was ensured by gentle restraint. Dogs were positioned in right and left lateral recumbency.

The following echocardiographic planes and parameters were evaluated:

- Right parasternal long- and short-axis views: assessment of global cardiac dimensions and structure.
- Right parasternal short-axis view at the level of the aortic valve: left atrial-to-aortic root ratio (LA/Ao).
- Right parasternal short-axis view at the level of the pulmonary artery: peak pulmonary artery flow velocity (PV).
- Left apical four-chamber view: early diastolic mitral inflow velocity (eV).
- Left apical four-chamber view with tissue Doppler imaging (TDI): septal and lateral mitral annular velocities (TDI-Sep E/Em and TDI-Lat E/Em).

Each measurement was performed in triplicate across three consecutive cardiac cycles, and the average was used for analysis. All procedures followed current veterinary cardiology standards and echocardiographic guidelines.

### 2.7. Statistical Analysis

Descriptive statistics were calculated to summarize baseline characteristics of both groups. Differences in PhA between PDA and control dogs were analyzed using independent samples *t*-tests. The effect of frequency (5, 50, and 250 kHz) on PhA was assessed using one-way analysis of variance (ANOVA) with post hoc testing. Changes in PhA before and after PDA ligation were analyzed using repeated-measures ANOVA, while paired *t*-tests were applied for pre- versus post-surgical comparisons of echocardiographic variables (e.g., LA/Ao, TDI E/Em). Group comparisons of independent variables were performed with unpaired *t*-tests.

The relationship between PhA and echocardiographic indices (eV, PV) was evaluated using Pearson’s correlation coefficient and linear regression analysis. Data normality was assessed with the Shapiro–Wilk test and visual inspection of QQ plots. A *p*-value < 0.05 was considered statistically significant.

## 3. Results

### 3.1. Regional Comparison of PhA Measurements

This analysis evaluated PhA across three anatomical regions—the thorax, trunk, and abdomen—in 13 dogs with PDA to assess the impact of electrode placement on impedance-derived measurements.

**Figure 3:**
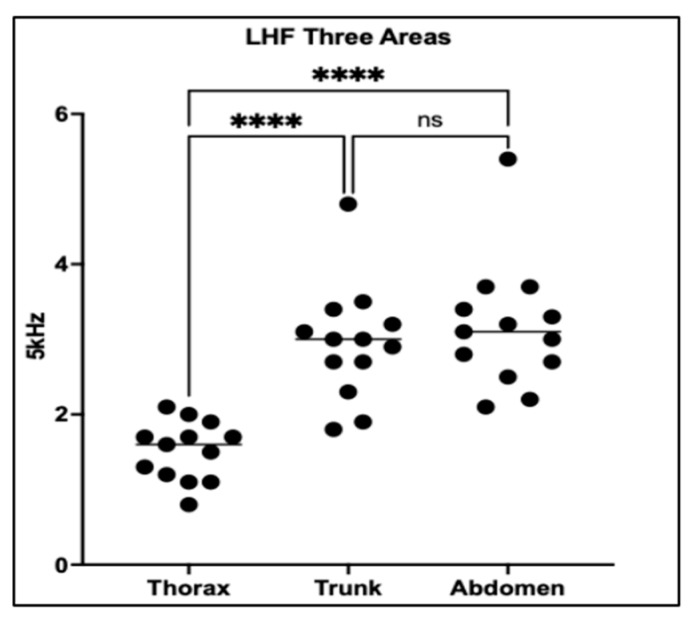
Scatter plots of PhA values obtained from thoracic, trunk, and abdominal regions in 13 dogs. Each dot represents an individual measurement. Thoracic PhA values were significantly lower than both trunk and abdominal measurements (*p* < 0.0001), while no difference was observed between trunk and abdomen (ns). These results highlight the enhanced sensitivity of thoracic measurements for detecting PDA-associated fluid imbalance localized to the chest. ****: *p* < 0.0001; ns: not significant.

One-way ANOVA followed by Tukey’s multiple comparisons test revealed significant regional differences in PhA (*p* < 0.0001). Thoracic PhA values were significantly lower than those obtained from both the trunk and abdominal regions (*p* < 0.0001 for both), suggesting that thoracic measurements are more sensitive to localized fluid accumulation associated with PDA. In contrast, no significant difference was observed between trunk and abdominal regions (*p* = 0.7110), indicating similar impedance properties at these sites (Figure 3).

### 3.2. Comparison of PhA Between PDA and Healthy Groups

A comparative analysis was performed on a subset of 15 dogs with Patent ductus arteriosus (PDA) and 15 healthy controls, with phase angle (PhA) measured at three frequencies (5 kHz, 50 kHz, and 250 kHz). Clear frequency-dependent differences were observed between groups.

At 5 kHz, dogs with PDA exhibited markedly lower PhA values compared with healthy controls (*p* < 0.0001), highlighting the high sensitivity of low-frequency PhA to extracellular fluid accumulation and volume overload associated with PDA. At 50 kHz, a significant difference between groups was also detected (*p* = 0.0125), although the magnitude of separation was less pronounced than at 5 kHz. This suggests that mid-frequency PhA retains some discriminatory capacity but may be less sensitive to the hemodynamic disturbances of PDA.

**Figure 4:**
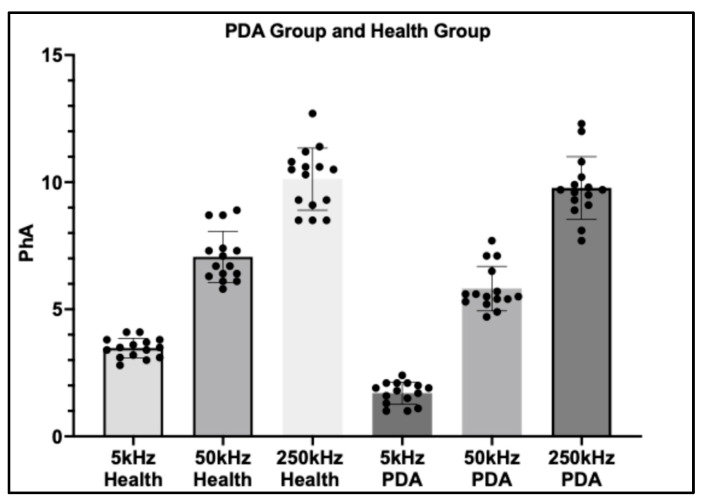
Bar chart illustrating PhA values at 5 kHz, 50 kHz, and 250 kHz in dogs with PDA compared with healthy controls. PhA at 5 kHz was markedly reduced in the PDA group, demonstrating its superior sensitivity to extracellular fluid imbalance. In contrast, differences at 50 kHz were less pronounced, and no significant variation was observed at 250 kHz. These findings highlight low-frequency PhA (5 kHz) as a promising non-invasive parameter for disease assessment and monitoring in PDA.

In contrast, at 250 kHz, no statistically significant difference was observed between groups (*p* = 0.6956), indicating that high-frequency PhA is largely unaffected by PDA-related thoracic fluid alterations. Collectively, these findings underscore the diagnostic potential of low-frequency PhA, particularly at 5 kHz, as a non-invasive indicator of fluid imbalance in dogs with PDA (Figure 4).

### 3.3. Postoperative Changes in Echocardiographic Parameters

Among the 20 dogs with paired pre- and postoperative echocardiographic assessments, surgical ligation of PDA resulted in consistent and favorable hemodynamic alterations. A significant reduction in the left atrial-to-aortic root ratio (LA/Ao) was observed following surgery, reflecting a decrease in left atrial volume load and pressure. Specifically, the LA/Ao ratio declined from a mean value of 1.440 to 1.201 (*p* = 0.0026), supporting effective relief of left-sided volume overload after ductal closure.

In addition, tissue Doppler imaging (TDI)-derived indices of diastolic function demonstrated downward trends postoperatively. The septal E/Em ratio decreased from 14.62 to 10.80 (*p* = 0.0053), indicating a significant reduction in left ventricular filling pressures. Similarly, the lateral E/Em ratio declined from 9.952 to 7.783, although this change did not achieve statistical significance (*p* = 0.2187).

**Figure 5:**
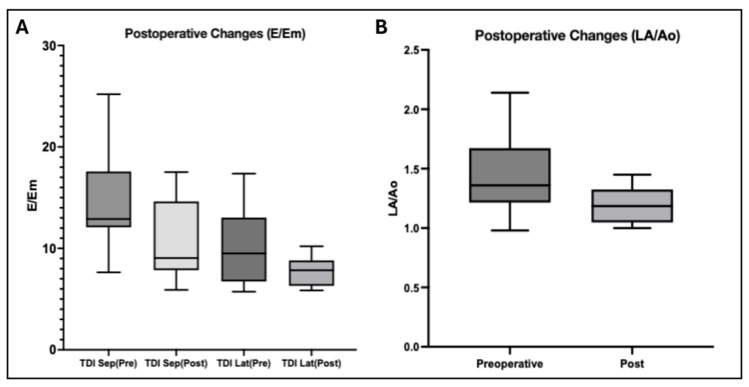
Echocardiographic changes before and after surgical correction of PDA. (**A**) Both TDI-derived diastolic indices, septal E/Em and lateral E/Em, demonstrated decreased median values and reduced variability postoperatively, reflecting lower left ventricular filling pressures and improved diastolic function. (**B**) The LA/Ao ratio showed a consistent downward shift after surgery, with reduced median and interquartile range, indicating a reduction in left atrial volume load and pressure.

Collectively, these echocardiographic findings confirm the hemodynamic benefits of PDA ligation, highlighting its role in alleviating left atrial pressure and improving ventricular diastolic loading conditions in affected dogs (Figure 5).

### 3.4. Pre- and Post-Surgical Changes in PhA

This analysis evaluated thoracic PhA at 5 kHz in 29 dogs diagnosed with PDA, with measurements obtained at multiple time points before and after surgical correction. The primary aim was to assess whether PhA could capture temporal changes in thoracic fluid redistribution following the elimination of left-to-right shunting.

**Figure 6:**
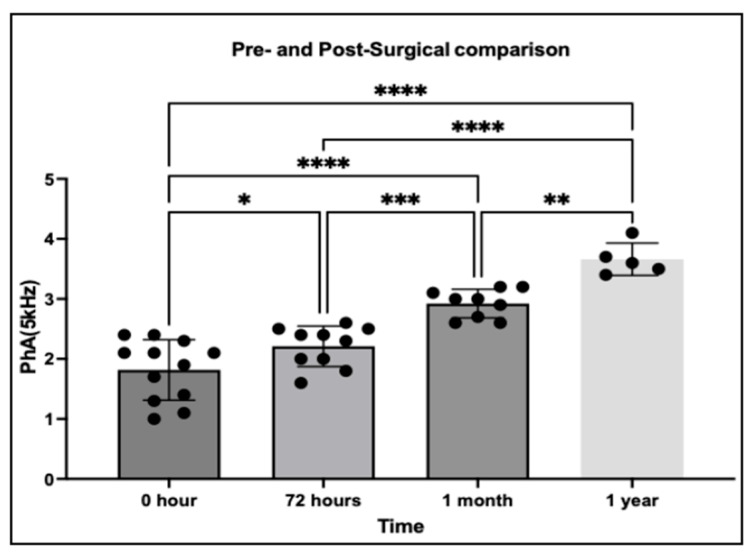
Pre- and postoperative changes in thoracic PhA at 5 kHz in PDA-affected dogs. A progressive increase in PhA was observed, with significant elevations at 72 h, 1 month, and 1 year after surgical correction. The results highlight PhA’s potential as a non-invasive biomarker for monitoring thoracic fluid redistribution and long-term recovery following PDA closure. ****: *p* < 0.0001; ***: *p* < 0.001; **: *p* < 0.01; *: *p* < 0.05; ns: not significant.

Following surgical correction, PhA values demonstrated a progressive increase over time, consistent with gradual improvement in thoracic fluid balance. One-way ANOVA confirmed significant differences among time points (*p* < 0.05), indicating a clear temporal trend in fluid redistribution. Compared with pre-surgical baseline, PhA was significantly elevated by 72 h postoperatively (*p* = 0.0209) and showed further significant increases at 1 month (*p* < 0.0001) and 1 year (*p* < 0.0001). These findings suggest that PhA reflects not only acute but also long-term improvements in thoracic fluid homeostasis following PDA correction (Figure 6).

### 3.5. Association Between Pulmonary Artery Velocity and Thoracic PhA

This analysis included 22 dogs with PDA in which both pulmonary artery flow velocity (PV) and thoracic PhA were available. The objective was to examine whether PhA, derived from bioelectrical impedance analysis (BIA), reflects pulmonary hemodynamic burden as indicated by PV.

Pearson’s correlation analysis demonstrated a strong inverse relationship between PV and PhA at 5 kHz (r = −0.6814, 95% CI: −0.8568 to −0.3645, *p* = 0.0005). A moderate negative correlation was observed at 50 kHz (r = −0.5077, 95% CI: −0.7655 to −0.1096, *p* = 0.0159), whereas no significant correlation was detected at 250 kHz (r = −0.2587, 95% CI: −0.6134 to 0.1829, *p* = 0.2451).

Linear regression analysis further confirmed these associations. At 5 kHz, PV significantly predicted PhA, with a regression slope of −0.01178 (95% CI: −0.01768 to −0.005876, *p* = 0.0005, R^2^ = 0.4643). At 50 kHz, the slope was −0.01322 (95% CI: −0.02367 to −0.002756, *p* = 0.0159, R^2^ = 0.2578). No significant predictive relationship was found at 250 kHz (slope = −0.01306, 95% CI: −0.03581 to 0.009688, *p* = 0.2451, R^2^ = 0.0669). Collectively, these results indicate that PhA measured at lower frequencies, particularly 5 kHz, is more sensitive to pulmonary overcirculation in PDA-affected dogs, supporting its potential role as a non-invasive biomarker for assessing hemodynamic burden.

### 3.6. Association Analysis Between End-Diastolic Volume and Thoracic PhA

This analysis evaluated the relationship between eV, a key indicator of cardiac remodeling, and thoracic PhA at multiple frequencies in 31 dogs with confirmed PDA.

Pearson’s correlation analysis demonstrated a moderate negative association between eV and PhA at 5 kHz (r = −0.5317, 95% CI: −0.7456 to −0.2185, *p* = 0.0021), indicating that higher eV values were associated with lower PhA. At 50 kHz, a weaker but still significant inverse correlation was observed (r = −0.4408, 95% CI: −0.6877 to −0.1025, *p* = 0.0131). No significant association was detected at 250 kHz (r = −0.1941, 95% CI: −0.5132 to 0.1720, *p* = 0.2953).

Linear regression analysis supported these findings. At 5 kHz, eV significantly predicted PhA (slope = −0.01348, 95% CI: −0.02164 to −0.005327, *p* = 0.0021), with 28.3% of the variability in PhA explained by changes in eV (R^2^ = 0.2827). At 50 kHz, the slope was −0.01496 (95% CI: −0.02652 to −0.003390, *p* = 0.0131, R^2^ = 0.1943). At 250 kHz, the relationship was not statistically significant (slope = −0.01262, 95% CI: −0.03684 to 0.01160, *p* = 0.2953, R^2^ = 0.0377). Collectively, these findings indicate that PhA at lower frequencies, particularly 5 kHz, is more sensitive to cardiac structural remodeling associated with PDA-induced volume overload, whereas higher-frequency PhA appears less responsive to such changes.

### 3.7. Association Between LA/Ao Ratio and Thoracic PhA

Data from 25 dogs with complete measurements of left atrial-to-aortic root ratio (LA/Ao) and thoracic PhA were analyzed to determine whether left atrial enlargement correlates with impedance-derived PhA. Pearson’s correlation indicated a weak to moderate positive relationship at 5 kHz (r = 0.3937, 95% CI: −0.0017 to 0.6827, *p* = 0.0515), which did not reach statistical significance. Correlations at 50 kHz (r = 0.2247, *p* = 0.2801) and 250 kHz (r = 0.2935, *p* = 0.1545) were weaker and non-significant. Linear regression analysis yielded a slope of 0.7606 at 5 kHz (95% CI: −0.0054 to 1.527, *p* = 0.0515, R^2^ = 0.1550), indicating that LA/Ao explains approximately 15.5% of the variance in PhA. Regression slopes at 50 kHz and 250 kHz remained non-significant (50 kHz: slope = 1.004, *p* = 0.2801, R^2^ = 0.0505; 250 kHz: slope = 2.203, *p* = 0.1545, R^2^ = 0.0861). These results suggest that LA enlargement has minimal influence on thoracic PhA.

### 3.8. Association Between TDI Septal E/Em and Thoracic PhA

Twenty dogs with PDA were analyzed to explore the relationship between septal diastolic function (TDI Sep E/Em) and thoracic PhA. Pearson’s correlation demonstrated no significant association at any frequency: 5 kHz (r = −0.0738, *p* = 0.7571), 50 kHz (r = 0.03083, *p* = 0.8973), and 250 kHz (r = −0.05993, *p* = 0.8018). Linear regression confirmed these findings, with slopes of −0.01034 (5 kHz, *p* = 0.7571, R^2^ = 0.00545), 0.009833 (50 kHz, *p* = 0.8973, R^2^ = 0.00095), and −0.02683 (250 kHz, *p* = 0.8018, R^2^ = 0.00359). These data indicate that septal diastolic function is not meaningfully associated with thoracic PhA in PDA-affected dogs.

### 3.9. Association Between TDI Lateral E/Em and Thoracic PhA

In the same cohort of 20 PDA dogs, lateral diastolic function (TDI Lat E/Em) was compared with thoracic PhA across frequencies. No significant correlation was observed at 5 kHz (r = 0.0054, *p* = 0.9821) or 250 kHz (r = −0.3105, *p* = 0.1827). However, at 50 kHz, a significant negative correlation was detected (r = −0.5227, 95% CI: −0.7839 to −0.1043, *p* = 0.0180), suggesting that lower PhA values may reflect impaired lateral myocardial relaxation. Regression analysis supported this finding, with a significant slope at 50 kHz (slope = −0.2512, 95% CI: −0.4540 to −0.0483, *p* = 0.0180, R^2^ = 0.2732), indicating that approximately 27.3% of the variation in PhA at this frequency can be explained by changes in TDI Lat E/Em. No significant associations were observed at 5 kHz (slope = 0.001129, *p* = 0.9821) or 250 kHz (slope = −0.2118, *p* = 0.1827). These results suggest that thoracic PhA at 50 kHz may modestly reflect variations in lateral diastolic function in PDA-affected dogs.

## 4. Discussion

Our study demonstrates that phase angle (PhA) is a useful marker of extracellular fluid imbalance in dogs with Patent ductus arteriosus (PDA). At 5 kHz, PhA was significantly reduced in PDA dogs and progressively increased after surgery, reflecting recovery of thoracic fluid balance. Measurements at 50 kHz showed moderate sensitivity, while 250 kHz values were largely unchanged, underscoring the importance of frequency selection in Bioelectrical Impedance Analysis (BIA).

Thoracic electrode placement (left axilla to the 13th thoracic vertebra) allowed region-specific evaluation of pulmonary overcirculation and left-sided volume overload [22,23]. Low-frequency PhA reflects extracellular fluid expansion and pulmonary congestion, supporting its role as a non-invasive marker of PDA severity and monitoring [8,24]. In contrast, higher frequencies (50–250 kHz) integrate intracellular compartments and are influenced by cell mass rather than extracellular volume [8,25]. These findings align with human studies, where low-frequency PhA is closely linked to extracellular water and volume overload [24]. Clinically, 5 kHz PhA may be a practical tool when combined with echocardiographic and biochemical markers.

The postoperative rise in thoracic PhA indicates normalization of extracellular fluid after hemodynamic stabilization. Significant increases appeared within 72 h, reflecting early unloading of the pulmonary vasculature. Continued elevation at 1 month and 1 year suggests sustained improvement, likely associated with reverse remodeling and vascular recovery. Similar patterns are seen in human cardiac patients, where PhA rebounds during recovery, paralleling fluid normalization [8,25,26,27]. These findings support thoracic PhA as a dynamic marker for monitoring therapeutic response and long-term recovery.

In dogs with PDA, the observed inverse relationship between pulmonary artery flow velocity (PV) and low-frequency PhA highlights the link between hemodynamic burden and thoracic fluid distribution. Elevated PV reflects increased left-to-right shunting, leading to left atrial and ventricular volume overload, elevated preload, and potential pulmonary venous congestion—hallmarks of PDA pathophysiology and precursors to congestive heart failure if untreated [3,28,29,30]. The strong negative correlation between PV and 5 kHz PhA indicates that low-frequency measurements are particularly sensitive to extracellular fluid accumulation induced by volume overload [8,31]. At 50 kHz, a weaker yet significant correlation was observed, likely due to the mixed contribution of intra- and extracellular compartments, whereas 250 kHz PhA, primarily reflecting intracellular water, showed no meaningful association with PV.

End-diastolic volume (eV) reflects the structural remodeling of the left ventricle in response to chronic volume overload, making it a key indicator of long-term cardiac adaptation in dogs with PDA [8,31,32]. In this context, the observed inverse relationship between eV and low-frequency thoracic PhA underscores the interplay between structural changes and fluid dynamics. Specifically, lower 5 kHz PhA values in dogs with larger eV likely reflect extracellular fluid accumulation secondary to persistent left-to-right shunting and pulmonary overcirculation.

The frequency-dependent nature of this association highlights the physiological specificity of BIA measurements. Low-frequency PhA, which primarily traverses extracellular compartments, appears particularly sensitive to volume overload, whereas high-frequency PhA, influenced more by intracellular water and cellular integrity, fails to capture these changes. This distinction suggests that PhA can provide dynamic, functional insights into extracellular fluid shifts that are not apparent from static anatomical measurements like eV [33].

The marked reduction in thoracic PhA compared to trunk and abdominal measurements emphasizes the regional specificity of impedance-based assessments in detecting PDA-associated fluid alterations [34]. In PDA, chronic left-to-right shunting elevates pulmonary blood flow and left atrial pressure, promoting pulmonary vascular congestion and localized extracellular fluid accumulation within the thoracic cavity [15]. By directly measuring electrical impedance across this region, thoracic PhA appears particularly sensitive to these pathophysiological fluid shifts. In contrast, trunk and abdominal PhA values showed no significant differences, likely reflecting the inclusion of heterogeneous tissue compartments where localized thoracic fluid changes are diluted. This highlights that broader impedance measurements may underestimate regionally concentrated fluid overload. These results underscore the importance of anatomically targeted electrode placement in BIA, as measurement sensitivity can vary with both the disease process and the regional distribution of fluid accumulation [15,35].

The weak or frequency-dependent associations observed between thoracic PhA and echocardiographic indices—including LA/Ao, TDI septal E/Em, and TDI lateral E/Em—likely reflect the different physiological aspects captured by these measures. While LA/Ao and E/Em ratios quantify structural remodeling and diastolic function under chronic volume overload, PhA derived from BIA primarily reflects extracellular and intracellular fluid distribution, cell membrane integrity, and tissue hydration status [36,37,38,39,40]. This distinction suggests that PhA offers complementary information on systemic and regional fluid dynamics rather than duplicating structural changes detectable by echocardiography.

The limited correlation of PhA with LA/Ao suggests that atrial enlargement alone does not directly alter impedance. Conversely, the negative correlation between TDI Lat E/Em and 50 kHz PhA implies that mid-frequency values may capture subtle interactions between myocardial relaxation and fluid shifts, possibly indicating early diastolic dysfunction. Unlike 5 kHz PhA (extracellular) and 250 kHz PhA (intracellular), the 50 kHz signal integrates both compartments, making it sensitive to nuanced changes in compliance and interstitial fluid [40].

These findings are consistent with reports in other canine cardiac diseases, such as myxomatous mitral valve disease, where reduced 50 kHz PhA is associated with congestion and elevated NT-proBNP [41]. Together, our results suggest that thoracic PhA is linked to both structural and functional alterations in PDA: eV reflects chronic remodeling, PV and TDI reflect hemodynamic load, and PhA bridges these indices by indicating regional fluid status.

The correlations observed in this study indicate that thoracic PhA is linked to both structural and functional alterations in PDA. Its association with eV reflects chronic remodeling of the left ventricle, while correlations with PV and TDI parameters point to sensitivity for hemodynamic load and diastolic function. These results suggest that PhA provides complementary information that bridges fluid dynamics with conventional echocardiographic evaluation.

Overall, the weak correlations with echocardiographic indices underscore the complementary nature of PhA. While LA/Ao and TDI remain central for structural and functional evaluation, PhA adds unique information on fluid distribution and cellular changes. Integrating these measures could improve the assessment of PDA severity, perioperative management, and long-term remodeling.

### Limitations

While this study highlights the potential of phase angle (PhA) as a non-invasive indicator of thoracic fluid status in dogs with Patent ductus arteriosus (PDA), several limitations must be acknowledged. PhA is influenced by body composition, hydration, and disease-related fluid shifts, and variability in volume overload or diuretic therapy may have introduced inter-individual differences. The modest sample size, particularly the small number of healthy controls, and limited population diversity may restrict generalizability and introduce selection bias. Hydration and nutritional status were not standardized, adding further variability. The modest associations between PhA and echocardiographic indices likely reflect physiological differences: TDI E/Em captures instantaneous relaxation, whereas PhA reflects broader integration of fluid distribution and cellular integrity. No a priori sample-size calculation was performed, as this exploratory study was constrained by case availability, which may limit statistical power. Moreover, threshold values for PhA in PDA remain undefined, restricting immediate clinical use. Postoperative changes in PhA may also be affected by medications, comorbidities, or individual remodeling, complicating interpretation. The lack of correlation between thoracic PhA and left atrial size further suggests that PhA alone cannot reflect atrial remodeling and should be interpreted alongside echocardiographic markers. Future studies should use larger, multi-center cohorts, standardized hydration and nutrition assessments, and longitudinal monitoring, and integrate PhA with echocardiographic and biochemical markers to validate its diagnostic role and establish reference ranges in veterinary cardiology.

## 5. Conclusions

This study highlights the potential of phase angle (PhA) at 5 kHz as a non-invasive marker of thoracic fluid imbalance in dogs with Patent ductus arteriosus (PDA). Reduced low-frequency PhA likely reflects pulmonary extracellular fluid from chronic volume overload, while its strong negative correlation with pulmonary velocity (PV) shows its value in assessing hemodynamic burden. The postoperative rise in PhA further supports its role in tracking fluid recovery and thoracic remodeling after surgery.

By providing real-time information on extracellular fluid, thoracic PhA may complement echocardiography and biochemical markers, offering a rapid and practical tool for evaluating volume status in PDA. Further studies with larger, more diverse cohorts are needed to confirm its relationship with left-sided congestion and to establish reference ranges for clinical use in veterinary cardiology.

## Figures and Tables

**Table 1:** Distribution of Canine Subjects and Inclusion in Specific Analyses. (Note: The number of dogs differs across analyses due to incomplete echocardiographic or bioimpedance data in some individuals.).

	Sample Size		
Analysis Type	PDA	Healthy	Total
Group Comparison (PDA vs. Healthy)	15	15	30
PV vs. PhA Correlation	22	0	22
eV vs. PhA Correlation	30	0	30
LA/Ao vs. PhA Correlation	24	0	24
TDI Sep E/Em vs. PhA Correlation	20	0	20
TDI Lat E/Em vs. PhA Correlation	20	0	20
Pre- and Post-Surgical Comparison	29	0	29
Regional PhA Comparison	13	0	13

**Table 2:** Drug Dosages for Induction and Maintenance of Anesthesia.

Drug Name	Recommended Dosage Range (mg/kg)	Route of Administration	Notes
Atropine	0.02–0.04	SC/IM/IV	Anticholinergic agent for heart rate control and salivation reduction
Buprenorphine	0.01–0.02	IM/IV	Partial μ-opioid receptor agonist; analgesia
Cefazolin	20–22	IV	Antibiotic; used for preoperative infection prophylaxis
Midazolam	0.2–0.3	IV/IM	Sedative and anticonvulsant
Succinylcholine	1–2	IV	Rapid-acting muscle relaxant for tracheal intubation
Propofol	4–6 (induction dose)	IV	Rapid induction of general anesthesia
Isoflurane	Inhaled (1–2%)	Inhalation	Used for maintenance of general anesthesia

## Data Availability

The data presented in this study are available on request from the corresponding author due to ethical restrictions related to clinical veterinary records.

## Abbreviations

The following abbreviations are used in this manuscript:

BIA	Bioelectrical Impedance Analysis
PDA	Patent ductus arteriosus
PhA	Phase angle
PV	Pulmonary Velocity
LHF	Left Heart Failure
eV	End-diastolic volume
